# Expanding the CRISPR/Cas toolkit: applications in proteomics and theranostics

**DOI:** 10.3389/fbioe.2025.1713700

**Published:** 2025-11-27

**Authors:** Ashwini Punde, Saurabh Dey, Riya Pandire, Arindam Bhattacharjee, Chinmoy Patra

**Affiliations:** Department of Developmental Biology, Agharkar Research Institute, Pune, India

**Keywords:** CRISPR-Cas system, genome-engineering, proteomics, protein-protein interaction, protein-chromatin interaction, theranostics

## Abstract

Conventional methods available for genome editing have proven non-specific, labour-intensive, and time-consuming. In this context, CRISPR/Cas technology represents a significant breakthrough. It is derived from a sophisticated microbial defence system consisting of clustered regularly interspaced short palindromic repeats, or CRISPR, and the RNA-guided DNA endonuclease Cas. Beyond its original role in genome editing, CRISPR continues to play a major role in the field of proteomics, functional genomics, and molecular therapy. Animal models, including mice, *Drosophila*, zebrafish, *etc.*, have substantially benefited from CRISPR in uncovering protein function through reverse genetics approaches, including knock-in, knockout, CRISPRi, and indel mutation strategies. On the clinical front, CRISPR gene therapy has also seen successes, including applications in sickle cell disease, hypercholesterolemia, and cancer immunotherapy. However, notable challenges remain, including *in vivo* packaging and delivery efficiency, toxicity, and genomic off-target effects. Ongoing efforts to overcome these include the development of novel delivery formulations (e.g., nanoparticles, exosomes), artificial intelligence-guided experimental design, and miniaturization of Cas proteins. This review focuses on CRISPR/Cas gene editing mechanisms and explores its state-of-the-art applications in the field of proteomics and theranostics.

## 1 Introduction

Genome editing has been a long goal in molecular biology, medicine, and biotechnology, dating back to the discovery of restriction enzymes. However, the identification of zinc-finger nucleases (ZFN) (Urnov et al., 2010), transcription activator-like effector nucleases (TALEN) (Miller et al., 2011), and more recently, the CRISPR/Cas system, which employs clustered regularly interspaced short palindromic repeats and nuclease(s), has spearheaded the genome editing era (Jinek et al., 2012).

DNA sequences can be precisely cleaved using these tools that trigger double-strand breaks (DSBs) in the target sequence (Mukherjee et al., 2020). Subsequently, repair mechanisms such as the error-prone non-homologous end joining (NHEJ) or the precise homology-directed repair (HDR) are used to repair damage, which leads to target-area insertions/deletions (indels), and substitutions (Gaj et al., 2013). Nonetheless, CRISPR provides several advantages over legacy editors, including simplicity of target design, the predictability of off-target sites, and the ability to modify multiple genomic sites simultaneously (multiplex editing). The two essential components of CRISPR-Cas systems include the guide RNA (gRNA) and the CRISPR-associated Cas protein. The gRNA is around 20-nucleotide-long gene-specific sequence. The Cas-gRNA complex binds to DNA close to the protospacer adjacent motif (PAM), and gRNA instructs the Cas nuclease to make a double-strand cut in the target sequence (Jiang et al., 2013), which incorporates indels after NHEJ repair. Apart from the widely used SpCas9 from *Streptococcus pyogenes*, newly emerging CRISPR/Cas systems like SpCas9-NG, base editors, xCas9, Cas12a, Cas13, and Cas14 have gained popularity (Xu and Li, 2020). Further, Cas9 nickase, a mutant version of Cas9, generates a single-stranded break at the gRNA target site instead of the wild-type Cas enzyme-mediated double-stranded DNA break (https://doi.org/10.1038/s41467-023-37507-8). Double nicking strategy on the opposite strand of DNA can be used to reduce off-target effects.

Due to its versatility and robustness, CRISPR has become an indispensable tool in the development of complex biological model systems (Pickar-Oliver and Gersbach, 2019), proteomics, and theranostics (combined therapy and diagnosis) (Vandemoortele et al., 2016; Li et al., 2023). In the field of proteomics, CRISPR has enabled the identification of protein interaction partners (Dalvai et al., 2015), gene loci associated with protein interaction (Fujita and Fujii, 2013), protein localization (Cho et al., 2022), and the development of cellular models to investigate the downstream proteome (Mehrabian et al., 2014). On the clinical front and especially theranostics, CRISPR has been extensively used towards rectification of detrimental base mutations, disruption of disease-causing genes *via* engineered gene knockouts, and insertion of point mutations in a gene to assess their functional importance (Li et al., 2023). However, the rapid progress is somewhat tempered by challenges intrinsic to the CRISPR mechanism. Firstly, genome-wide off-target effects of the CRISPR/Cas system, which relies on the binding of a single gRNA, remain a major concern due to PAM-adjacent flexibility, which remains a drawback against the historically more precise ZFN/TALEN systems, whose working principle depends on the binding of two TALEN or ZFN arms on the opposite strand of the DNA in close proximity. However, recent discoveries showed that the application of two gRNAs targeting opposite strands at close proximity, along with Cas9 nickase, could reduce the off-target effects (Klermund et al., 2024). Second, the reliance on error-prone repair pathways produces a heterogeneous mutation spectrum. Third, the immunogenicity of bacterial Cas proteins and delivery systems such as AAV remains a challenge. Fourth, the sheer size of Cas variants limits their efficient delivery (Wang Y. et al., 2024). Engineered compact Cas variants, non-viral delivery systems, and AI-assisted guide design are some of the ways these challenges are being addressed.

Of recent, reviews on CRISPR have generally become narrowly specialized, often lacking a comprehensive synthesis that connects the naturally interrelated domains of proteomics, diagnosis, and therapeutics. This review, therefore, summarizes the gene editing mechanisms of CRISPR, followed by a discussion on its recent technological advances in these fields, highlighting current challenges and potential mitigation strategies.

## 2 CRISPR/Cas genome editing mechanisms

**FIGURE 1:**
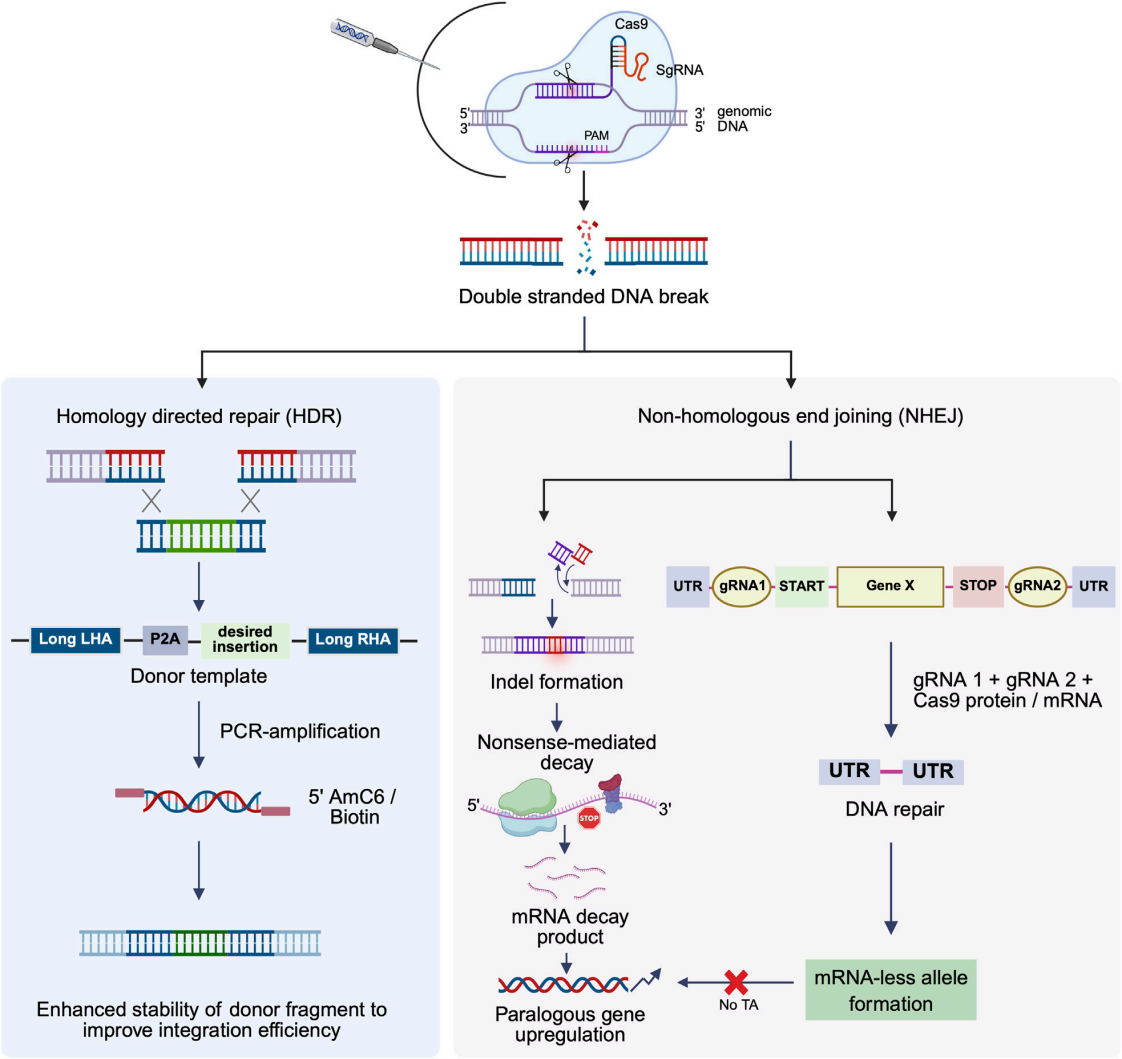
Schematic representation of the optimized CRISPR/Cas9 strategy for gene mutation, knockout, and enhanced knock-in efficiency. This figure depicts the process by which the CRISPR-Cas9 system introduces targeted genetic modifications. The Cas9 nuclease, guided by a single-guide RNA (sgRNA), generates a double-stranded break (DSB) at a specific genomic locus. Following cleavage, the cell activates one of two primary endogenous DNA repair pathways: non-homologous end joining (NHEJ) or homology-directed repair (HDR), resulting in different gene editing outcomes. NHEJ directly ligates the broken DNA ends without a template, often resulting in small insertions or deletions or in combination (indels) that can disrupt gene function. Dual gRNA-mediated knockout generates mRNA-deficient alleles, thereby effectively preventing transcriptional adaptation (TA) in knockout models (right). In contrast, HDR utilizes an exogenously supplied donor DNA template with homology arms flanking the break site to introduce precise sequence changes. 5′ modification of donor templates with amino (AmC6/12) or biotin linkers enhances template stability and increases the efficiency of targeted integration. This pathway facilitates precise gene correction, targeted mutagenesis, and transgene insertion. TA: transcriptional adaptation.

Briefly, the CRISPR/Cas9 genome editing mechanism is divided into three steps: recognition, cleavage, and repair (Shao et al., 2016). The specificity of the Cas9 nuclease is determined by the ∼20 nt guide sequence within the sgRNA. The sgRNA directs Cas9 to the target sequence in the gene of interest *via* its 5′ complementary base pairing with the crRNA (target-specific sequence within the sgRNA). The routinely used *S. pyogenes* Cas9 recognizes the PAM sequence at 5′-NGG-3′ (where N can be any nucleotide) and cleaves the DNA 3 base pairs upstream of the PAM. Cas9 contains two nuclease domains–the HNH domain cleaves the complementary strand of the target DNA at the localized site, while the RuvC domain cleaves the non-complementary strand, together leading to the formation of double-stranded breaks (DSBs) with blunt ends. The double-strand breaks at the target locus activate cellular DNA repair, resulting in two types of genome modifications: constitutive knockouts (KO) *via* NHEJ, and knock-in (KI) *via* HDR (Figure 1) (Sun et al., 2022).

### 2.1 CRISPR/Cas delivery systems

Cas9, along with sgRNA, can be delivered as DNA, mRNA, or as ribonucleoproteins. In tissue culture, packaging is done in a single chimeric SpCas9-gRNA plasmid such as PX330 (Cong et al., 2013). *In vivo* gene therapy relies on packaging of these components in viral or non-viral delivery systems, including adeno-associated virus (AAV; smaller packaging capacity of <5 kb), lentivirus (∼10 kb packaging capacity but higher propensity of genomic integration), and as nanocarriers such as packaged lipid nanoparticle (LNP) co-encapsulating both Cas9 mRNA and sgRNA.

Circumventing the payload limitations, immunogenicity, and toxicity of viral methods, non-viral delivery systems, such as nanoparticles (NPs), have been developed (Table 1). These offer distinct advantages, such as non-integration into the genome, resulting in significantly fewer off-target effects, and low immunogenicity due to their complete synthetic nature (Raguram et al., 2022). NP delivery can be tuned to be sensitive to pH, light, redox, or magnets, thus offering precise targeting to its cellular milieu (e.g., pH-based guidance in an acidic tumour microenvironment, photosensitive delivery, and external magnetic guidance). Other nanocarriers have been explored as well–such as organic (PEG, PEI, chitosan) and inorganic NPs (gold, silica), and DNA origami nanostructures. Crosslinked inorganic nanoparticles can be engineered to adsorb cargo to the surface lattice, including ssDNA donors and Cas9 (Duan et al., 2021), while minimizing toxicity. Meanwhile, DNA origami nanostructures–self-assembled tubules that internalize Cas9/sgRNA, offer high biocompatibility and have shown promise in gene therapy (Tang et al., 2023). Furthermore, a hybrid strategy such as LNP-SNA (lipid nanoparticle-spherical nucleic acid), structurally an LNP core with a spherical DNA shell, greatly improves both efficacy and biocompatibility of LNPs (Han et al., 2025). Efficacy of LNPs is further constrained to tissues such as the liver, due to their endocytic route, limited biodistribution (Yan et al., 2022), and low endosomal escape (i.e., propensity for endolysosomal degradation (Chatterjee et al., 2024). In general, tunable hybrid nanoplatforms are gaining popularity for the delivery of CRISPR components (Cavazza et al., 2025).

**TABLE 1:** Current *in vitro* and *in vivo* delivery methods for CRISPR components.

Delivery method	Delivery method subtype	Description	Advantages	Disadvantages	References
Viral vector	AAV, lentivirus, etc.	Engineered viruses to carry DNA encoding Cas enzyme + guide RNA	High transduction efficiency; well-studied; can reach multiple cell types *in vivo*.	Risk of genomic integration; payload size limitation (e.g., 4.7 kb for AAV); immune response upon repeated exposure, persistent expression may increase off-target risk.	Alves et al. (2025)
Physical	Electroporation, microinjection	Use of physical means to deliver CRISPR components	Very direct; microinjection can approach ∼100% in single cells; suitable for *ex vivo*.	Often low efficiency in hard-to-transfect cells, high cell stress or toxicity; may not work well *in vivo* for deep tissues.	Karp et al. (2025), Mahdi et al. (2025)
Nanoparticle	Lipid nanoparticle	Synthetic carriers (lipids, polymers, metals) to encapsulate CRISPR components and deliver via endocytic uptake	Lower immunogenicity; transient editing (less off-target); highly tunable carriers and guided targeting (size/composition/pH/light).	Delivery efficiency *in vivo* is still often lower than viral in many cases; targeting specificity is limited to a few tissues; low endosomal escape, i.e., prone to lysosomal degradation.	Hołubowicz et al. (2024), Musunuru et al. (2025)
	Non-lipid nanoparticle		Target cell specificity can be achieved by biomimetic metal organic frameworks (e.g., zeolitic imidazolate)	Toxicity, bioavailability	Alyami et al. (2020), Duan et al. (2021), De Carli et al. (2025)
Nucleic acid-based	DNA origami nanostructure	Engineered DNA nano-objects that bind CRISPR components	Vastly reduced toxicity, ease of designing via rolling circle amplification	Still in a very early stage; mostly *in vitro*; likely to address scaling, stability, and immunogenicity questions; yet to be proven broadly *in vivo*.	Tang et al. (2023)
	Hybrid DNA-lipid nanostructures	Spherical nucleic acid- LNP conjugate (SNA-LNP)	Enhanced cellular uptake compared to LNPs, high biocompatibility	Limited data for efficacy as of present	Han et al. (2025)
Small vesicles	Exosomes	Naturally derived (e.g., secretory) vesicles produced by cells	High biocompatibility, low immunogenicity, able to cross the blood-brain barrier, and preferential targeting of cancer cells	Currently, low loading efficiency; heterogeneity of EVs make reproducibility a challenge; scaling up production and targeting efficiently remains difficult.	Ilahibaks et al. (2024), Ye et al. (2025)
	Enveloped delivery vehicles	Viral origin particles which package CRISPR RNPs	No genome integration, easy cell penetration by membrane fusion	Immune response, loading efficiency (similar to viral vectors)	Karp et al. (2025), Xu et al. (2025)
Peptide-based	Cell penetrating peptide (CPP)	CRISPR components conjugated to peptides that facilitate membrane penetration	Very low toxicity; able to cross the blood-brain barrier, can enhance the cell penetration of an existing carrier	Prone to degradation *in vivo*; detection is challenging because of the size limit of conjugated tags, antibodies, etc.	Guzman Gonzalez et al. (2024), Wang et al. (2024a)

In summary, while viral vector systems currently excel in delivery efficiency, nanomaterials offer great fine-tuning in terms of packaging, delivery, and bioavailability, along with reduced toxicity and immunogenicity. Very recently, AI-guided design of novel NP formulations, prediction of kinetics, and biocompatibility have taken shape (Ruiz-Escudero et al., 2025; Zhang Z. et al., 2025), although their CRISPR and gene therapy-specific applications remain unexplored. Presently used CRISPR delivery methods are highlighted in Table 1.

### 2.2 CRISPR/Cas-mediated gene knockout

Taking advantage of the error-prone nature of NHEJ repair, researchers widely use it to study gene loss-of-function in fields including functional genomics, reverse genetics, disease modelling, and small molecule screening for drug discovery (Gurumurthy et al., 2016; Neggers et al., 2018; Zarei et al., 2019; Wang and Doudna, 2023). Frameshift mutations introduced by a single gRNA within the coding sequence of a gene often result in premature termination codons (PTCs), leading to the production of truncated, non-functional proteins. However, mRNAs containing PTC are typically recognized and degraded by nonsense-mediated mRNA decay (NMD) (Kurosaki et al., 2019; Monaghan et al., 2023). This can activate genetic compensation or even an aggregated rescue phenotype by “transcriptional adaptation (TA),” i.e., through transcriptional upregulation of genetic or functional paralogs (Rossi and Gonzalez, 2015; El-Brolosy et al., 2019; Serobyan et al., 2020). This can be circumvented by using dual gRNAs targeting sequences flanking the entire gene locus, or by deleting the promoter sequence of the gene (Kumari et al., 2022). This strategy prevents any genetic rescue by preventing the formation of truncated mRNA, effectively bypassing NMD.

### 2.3 Site-directed knock-in using CRISPR/Cas

Cas9-induced double-strand breaks can trigger HDR, facilitating precise gene knock-in. To achieve a knock-in, a donor template must be introduced, consisting of the desired sequence flanked by homology regions corresponding to the area on either side of the cut. Donor templates can be small (<200 nt) single-strand oligo deoxynucleotides (ssODN), which are ideal for small substitutions/insertions, single-strand DNA (ssDNA), for larger edits up to 1 kb, and dsDNA for edits >1 kb, such as endogenous large epitope tags. Gene knock-ins are more challenging than knockouts due to the lower prevalence of HDR than NHEJ, the size limitation of donors, and the potential off-target integration of larger templates. As a remedy, a hybrid ss-overhang containing dsDNA template (overhang double-stranded DNA or odsDNA) has shown a promising outcome for larger insertions (Han et al., 2023). CRISPR knock-in has been successfully used in several model organisms by us and others, including *Drosophila* (Bhattacharjee et al., 2022), zebrafish (Irion et al., 2014; Hisano et al., 2015; Mi and Andersson, 2023), and mice (Shen et al., 2013; Platt et al., 2014). More recently, Cas12a knock-in mice have been developed for multiplexing of gene editing and efficient pooled CRISPR screens (Tang et al., 2025). In recent years, CRISPR/Cas9-mediated knock-in technology has gained significant traction in the zebrafish research community for generating human disease models and inserting fluorescent reporters to study endogenous gene expression (Hruscha et al., 2013; Hwang et al., 2013; Tessadori et al., 2018; Mi and Andersson, 2023).

The efficiency of HDR is influenced by several characteristics of the donor fragment, including its availability, type, structure, and length (Liao et al., 2024). Notably, donor fragments modified at the 5′ end, such as with 5′AmC6 or 5′AmC12, have been shown to enhance knock-in efficiency across different cell types and model organisms (Yu et al., 2020; Ghanta et al., 2021; Mi and Andersson, 2023). More recently, 5′ biotinylation of dsDNA donors, combined with NHEJ suppression, has been shown to minimize off-target integrations and further enhance the precision of HDR-mediated insertions (Takagi et al., 2024).

## 3 Applications of the CRISPR/Cas system

Being a powerful and multiplexable genome editing technique, CRISPR has widely enabled precise gene alteration and, therefore, functional studies (Wang Y. et al., 2013; Jacinto et al., 2020; Ansori et al., 2023; Khan et al., 2025). Numerous genetically modified mice, zebrafish, *Drosophila*, *Bombyx mori*, *Caenorhabditis elegans*, crops, bacteria *etc.*, have been generated to elucidate gene functions, protein interactions, subcellular localization, and disease-associated signalling pathways (Friedland et al., 2013; Gratz et al., 2013; Wang H. et al., 2013; Dickinson and Goldstein, 2016; Baci et al., 2021; Ansori et al., 2023; Sun et al., 2024).

**FIGURE 2:**
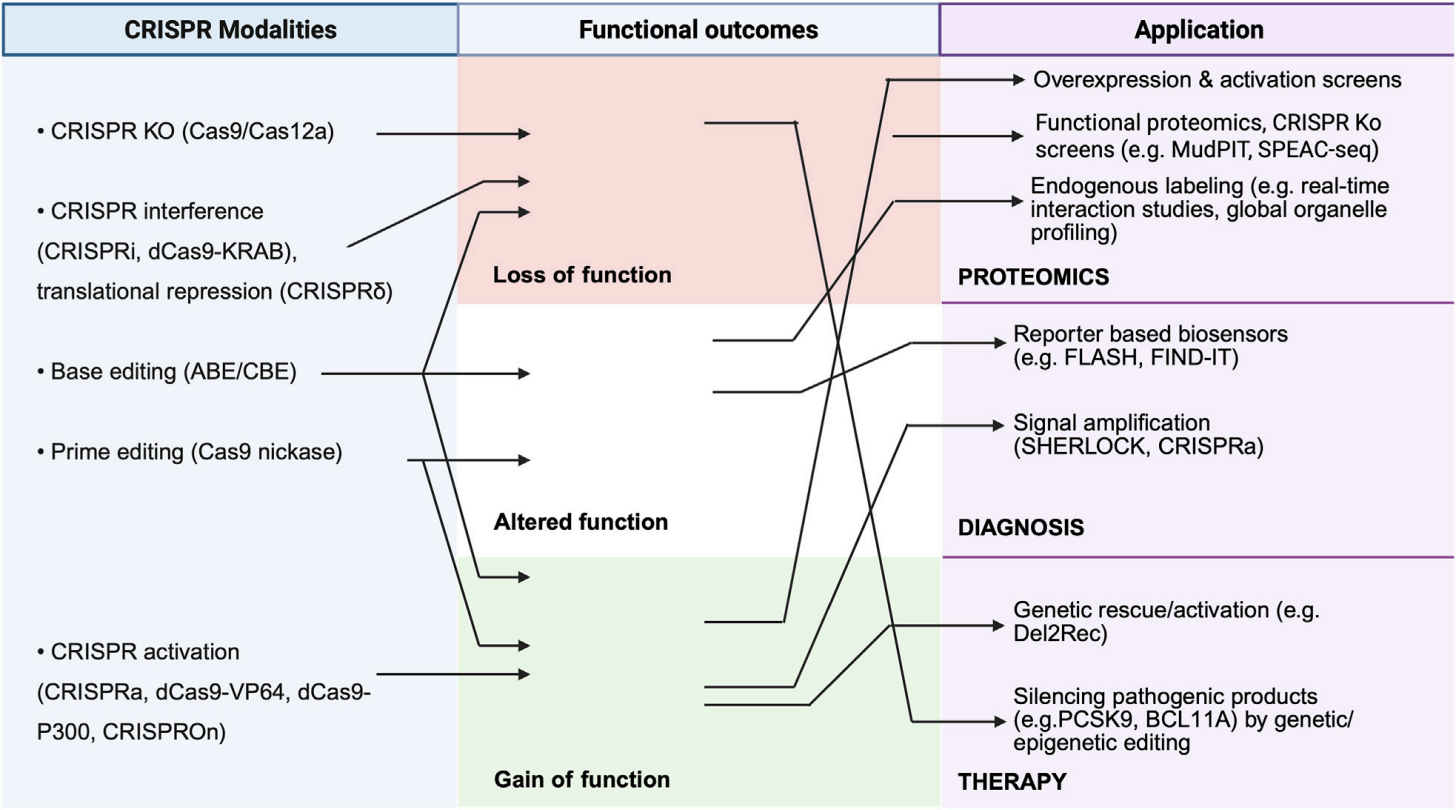
Mechanistic matrix of CRISPR functional outcomes across proteomics, diagnostics, and therapy. This figure depicts CRISPR modalities, including knockout, interference, epigenetic silencing, base/prime editing, and activation (left), enabling precise modulation of gene expression and genomic loci (middle). These approaches support diverse applications, such as endogenous tagging, reporter- and signal–amplification–based detection of foreign nucleic acids (e.g., DETECTR, SHERLOCK, SARS-CoV-2 assays), and functional proteomics through mapping protein–protein and protein–DNA interactions (right).

Applications of CRISPR has also found in theranostics, including diagnosis, genome editing, gene therapy, drug discovery, and epigenome editing (Figure 2). Advances in proteomics have enabled CRISPR-based diagnostic and therapeutic approaches. For example, a pan-cancer proteogenomics approach utilizing an integrated CRISPR dataset across >1,000 tumour samples (DepMap) has uncovered druggable targets based on their hyperactivation in tumour (Savage et al., 2024). The approach also helped to identify *bona fide* neoantigens (antigens absent in the normal human genome and therefore attractive targets). Another example is Systemic Lupus Erythematosus (SLE), an enigmatic autoimmune disease characterized by heterogeneous symptoms and an unpredictable prognosis. Here, CRISPR activation of miR-146a, whose downregulation is a key node in SLE progression, was used as a screen to identify SLE-linked functional enhancers of miR-146a, paving the way for therapeutic targeting (Zhu et al., 2024). Thus, mapping of protein expression, modification, and functional dependencies promotes the discovery of novel biomarkers, biomarker-based patient selection, and drug repurposing. This section discusses the applications of the CRISPR/Cas system in proteomics and modern theranostics.

### 3.1 CRISPR in proteomics

The development of proteomics is significantly aided by genome-editing technology. Currently, there are three different divisions through which CRISPR/Cas applications in proteomics have been divided: (i) the investigation of protein-protein interactions, (ii) protein-chromatin interactions, and (iii) the development of biological models. In most cases, CRISPR-Cas has acted as a bridge to existing techniques for investigating protein-protein and protein-chromatin interactions. Conversely, developing cellular models entails rapid and efficient gene editing to investigate gene function at the endogenous level.

#### 3.1.1 Protein-protein interactions

Protein-protein interaction (PPI) is an essential process in all living organisms that governs various regulatory cellular functions and signalling pathways. The manipulation of PPIs is of great importance in comprehending the molecular foundation of diseases and the development of therapeutics. Recently, CRISPR has emerged as a robust tool for exploring PPIs by simplifying endogenous tagging of target proteins for unbiased mapping of the interactome. In addition, approaches for real-time monitoring of protein dynamics, interaction, and characterization through targeted mutation of interacting amino acid residues or motifs have also gained attraction.

Stein et al. utilized Stable Isotope Labelling in Cell Culture (SILAC) and Multidimensional Protein Identification Technology (MudPIT) to compare interatomic profiles of the AMPKa2 subunit of the AMP-activated protein kinase. This study employed two different methods for protein isolation: CRISPR-based tag insertion followed by affinity purification and direct immunoprecipitation (Stein et al., 2019). In another study, the interactome of essential *T. gondii* proteins has been carefully examined using biotin identification (BioID) and CRISPR-Cas9. Through CRISPR-Cas9 knockdown and auxin-induced degradation, numerous proteins crucial for *Toxoplasma gondii* growth and invasion were discovered. CRISPR-based knock-in has been employed to introduce BirA biotin ligase into the genome, producing endogenous fusions of BirA with the gene of interest, followed by mass spectrometry investigation of interaction partners (Long et al., 2017b; 2017a). Another state-of-the-art technique termed SPEAC-seq offers direct, unbiased identification of factors regulating cell-cell crosstalk without *a priori* knowledge of a cellular BirA target. The molecular crosstalk between astrocytes and microglia, two glial subtypes that coordinate cytokine secretion and neuronal survival, was established this way (Wheeler et al., 2023). Here, the authors harnessed a CRISPR-KO library to generate single-cell KO microglia, which are individually co-cultured with a reporter astrocyte in picolitre droplets. Any CRISPR perturbation that modifies cell-cell crosstalk is registered as a reporter response. Library preparation and sequencing of sorted reporter-positive/negative droplets identified genes regulating astrocyte-microglia crosstalk. The technique could be extended to include different cell types and epigenetic perturbations to enhance understanding of cell-cell crosstalk (Faust Akl et al., 2025).

CRISPR is widely used to introduce targeted point mutations within gene regions encoding protein–protein interaction domains. Such mutations can disrupt or modify these interactions, thereby revealing the specific domains critical for functional association between the proteins (Després et al., 2020; Wang Y. et al., 2022). This strategy opens up new opportunities for drug development by screening for possible interactions with targeting medications. Additionally, fusion proteins, which unite two interacting proteins into a single molecule, may be created using CRISPR (Després et al., 2020), revealing clues about spatiotemporal protein interaction and kinetics.

Chong et al. inserted fluorescent tags into an interacting partner protein using a CRISPR-mediated knock-in strategy (Chong et al., 2015). Using this method, real-time interaction was observed, and information about its dynamics and subcellular localization was identified. Information about the localization of 1,311 human proteins encoding CRISPR-edited fluorescent tags has been incorporated in the previously mentioned OpenCell dataset, which was made feasible by automating image acquisition and manually analysing protein localization (Cho et al., 2022). Such endogenous tagging can solve unique challenges, e.g., a study used CRISPR-based fluorescent multiplexing of all known GABARAP family homologs, which play a crucial role in autophagy; however, they are difficult to distinguish by traditional immunostaining methods (likely possessing overlapped epitopes). The authors demonstrated that different GABARAP proteins interacted with a distinct population of autophagosomes, thus delineating their functional niche (Goldin-Azulay et al., 2024). Extending this approach globally, proteomic profiling of the total subcellular organelle population has been performed. Endogenous CRISPR tagging of organelle markers for native IP of organelles (to circumvent marker overexpression, which often mislocalizes) and cluster-based analysis of their proteome revealed important cellular context of co-functioning proteins in multiple related organelles (e.g., early and late endosomes in the endocytic pathway), organelle remodelling during viral infection, etc (Hein et al., 2025).

In conclusion, the use of CRISPR-based systems to examine protein-protein interactions has provided an effective tool for investigating the underlying biology of these interactions. Further studies are expected to uncover new opportunities for drug development by creating knockout cell lines, adding point mutations, creating fusion proteins, adding fluorescent tags, and other modifications.

#### 3.1.2 Protein-chromatin interactions

A multitude of biological activities, including the regulation of gene expression, depend on protein-chromatin interactions (PCIs). Histones, transcription factors, and chromatin remodelers are proteins that interact with chromatin, modifying its shape and function (Zhang et al., 2016). CRISPR-based PCI studies have contributed to the understanding of the molecular basis of gene regulation and disease pathogenesis.

Nuclease dead Cas9 (dCas9) can bind to the target DNA sequence directed by the gRNA, but cannot cause a double-stranded or single-stranded break. To modify gene expression or chromatin structure, dCas9 can be fused to various effector domains, including transcriptional activators, repressors, or epigenetic modifiers. CRISPR-dCas9 may be used for chromatin imaging, targeted epigenetic editing, and transcriptional control (Duke et al., 2020; Karlson et al., 2021). The DNA-targeting capabilities of dCas9 are used in several methods for studying locus-specific proteomes. Chromatin-associated proteins may be extracted and examined by adding an affinity tag to dCas9, followed by affinity purification of the dCas9-chromatin complexes from cells. To isolate the specific locus, dCas9 linked to a 3xFLAG tag is employed in the approach known as engineered DNA-binding molecule-mediated chromatin immunoprecipitation (enChIP), coupled to mass spectrometric identification of PCI (Fujita and Fujii, 2013). Later, the same group modified enChIP for prokaryotes (Fujii and Fujita, 2022). In a streamlined version of this strategy, a purified dCas9-3xFLAG-sgRNA RNP complex was added to cells after chemical crosslinking and chromatin shearing, which was directly used for affinity purification. Termed as Cas9 locus-associated proteome or CLASP (Tsui et al., 2018), this strategy can be rapidly adapted to any locus, as it only requires modifying the sgRNA sequence. In another study, a technique known as CRISPR-based chromatin affinity purification with mass spectrometry (CRISPR-ChAP-MS) was used, utilizing protein A as an affinity handle (Waldrip et al., 2014). The CRISPR affinity purification *in situ* of regulatory elements (CAPTURE) method is another method of this type that uses biotinylated dCas9 (Liu et al., 2017).

Proximity labelling may be used to identify the proteins linked to a specific genomic locus (Ummethum and Hamperl, 2020). In one such case, dCas9 and the BirA enzyme were combined to create a system called CasID. This approach was evaluated on genomic areas that have been well-researched, such as telomeres and large satellite repeats (Schmidtmann et al., 2016). The protein makeup of human telomeres and centromeres was investigated using the C-BERST (dCas9-APEX2 Biotinylation at Genomic Elements by Restricted Spatial Tagging) (Gao et al., 2018), and CAPLOCUS (combining CRISPR and the peroxidase APEX2 to identify local protein-chromatin interactions) (Qiu et al., 2019).

Other popular applications of CRISPR for studying the PCI include CRISPR-Targeted DNA Methylation Editing (CRISPR-TDM) and CRISPR-Targeted Histone Modification Editing (CRISPR-THME). Utilizing the CRISPR-Cas system, the CRISPR-TDM method accurately modifies DNA methylation patterns at specific genomic locations. To add or remove DNA methylation, the dCas9 is coupled to a DNA methyltransferase or a demethylase. CRISPR-TDM may be used to investigate how chromatin structure and gene expression are impacted by DNA methylation (Vojta et al., 2016; Katayama et al., 2021). CRISPR-THME, in contrast, makes use of the CRISPR-Cas system to precisely manipulate histone modifications at particular genomic loci, fusing dCas9 to histone-modifying enzymes, such as acetyltransferases or deacetylases (Pulecio et al., 2017; Syding et al., 2020).

### 3.2 Development of biological models

The development of biological models that faithfully replicate the *in vivo* setting is crucial for understanding the complex mechanisms underlying cellular functions and diseases. By comparison, traditional cell culture models often lack the complexity and physiological significance of the *in vivo* environment, resulting in a limited comprehension of the underlying processes. Through the precise and effective manipulation of specific genes and pathways made possible by CRISPR-based approaches, the construction of cellular models has undergone an unprecedented transformation, producing more accurate and relevant models. In addition, the ease and precision with which the CRISPR/Cas system allows for gene editing have revolutionized the field of genetic engineering, making it possible to create cellular models with unparalleled accuracy.

The existing paradigm for the initial evaluation of gene function is loss-of-function experiments (Syding et al., 2020). The implications of a target gene can be extensively examined, and the importance of the gene and its relationship to the phenotype can be clarified by creating a cell line with a deletion of the target gene. For these sorts of investigations, proteomics has been shown to be essential. Proteomics, combined with RNA interference, has already helped characterize the critical functional properties of several protein-coding genes (Brioschi and Banfi, 2018). Mehrabian et al. employed mass spectrometry to examine the phenotypic impact of the gene deletion in NMuMG epithelial cells after creating prion protein (PrP) knockouts using Cas9 (Mehrabian et al., 2014). *Escherichia coli* metabolic processes have been explored using CRISPR inhibition and quantitative mass spectrometry. Furthermore, specific mutations brought on by CRISPR-Cas base editors (Cas9 ‘nickases’ that edit single bases without resorting to a repair template) have also been investigated using proteomics. Landberg and colleagues employed CRISPRi in *E. coli* to silence a few essential genes involved in purine and pyrimidine biosynthesis (Landberg et al., 2020). The genes that were downregulated were subjected to proteomics analysis. Using a similar strategy of CRISPRi screening in combination with quantitative mass spectrometry, another team investigated the mechanisms that counteract the downregulation of numerous crucial enzymes in *E. coli* (Donati et al., 2021). CRISPR KO can be harnessed to identify genetic modifiers of a disease phenotype. In one such example, the widely used Huntington’s Disease (HD) mice model *Htt* ^*Q111*^ was subjected to CRISPR KO of probable candidate genes that could modify the number of CAG repeats in *Htt*, which is directly correlated with HD severity. The study identified several mismatch repair genes as CAG expansion modifying candidates (Mouro Pinto et al., 2025).

In addition to studies that include loss-of-function, significant disease-relevant models can be created using specific mutations that result in a gain-of-function impact. These mutations cause notable phenotypic alterations and are easily investigated using proteomics techniques. For instance, utilizing CRISPR-Cas genome editing with HDR, researchers introduced the EGFR C797S mutation, which is frequent in non-small-cell lung cancer, and examined its impact using differential proteomics and transcriptomics (Wang et al., 2020). Similar strategies have been used to generate animal models of short tandem repeat (STR) diseases such as HD and myotonic dystrophy by appending STR insertions to respective disease genes (Nutter et al., 2019).

A combination of quantitative mass spectrometry and CRISPR-Cas base editors has been employed. In one study, Chang et al. compared the HDR-based approach with base-editing to correct the G2019S mutation in leucine-rich repeat kinase 2 (LRRK2), a common genetic cause of Parkinsonism (Chang et al., 2021). They found that adenosine base editing using the ABEmax system had a higher percentage of correctly edited clones and a lower rate of off-target mutations than the HDR-based approach (Koblan et al., 2018). Phenotypic changes were compared using RNA-sequencing and mass spectrometry, and proteomics was found to be more consistent with the observed phenotype and data from previous studies.

Apart from enabling the study of the effects of specific gene mutations on cancer development and progression, leading to the identification of novel therapeutic targets (Liu et al., 2019), CRISPR-based cellular models have also been used to study the effects of specific epigenetic modifications on neuronal development and function, providing insights into the mechanisms of neurological disorders (Day, 2019; Kampmann, 2020).

### 3.3 CRISPR in theranostics

**FIGURE 3:**
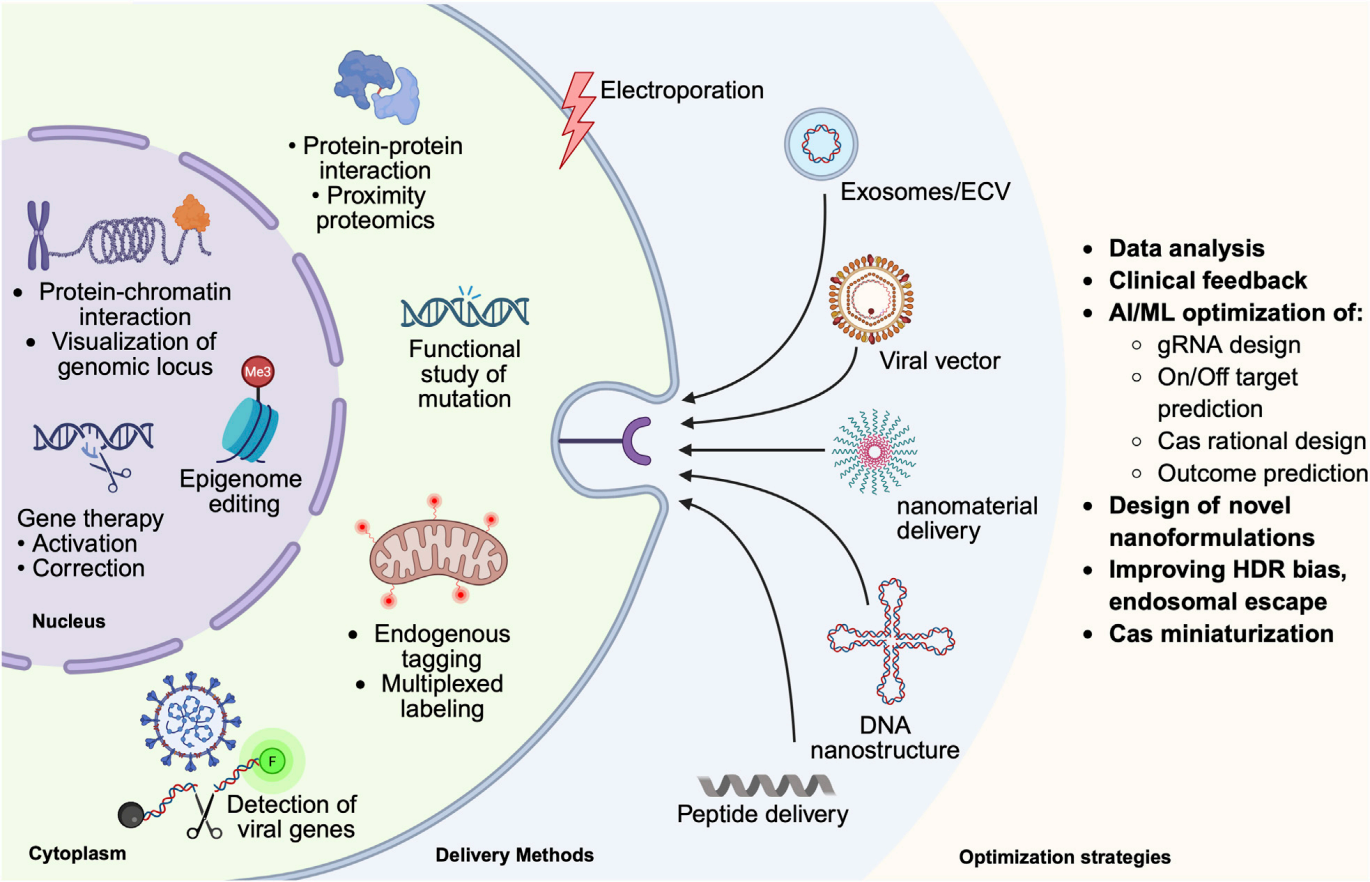
Integrated proteomics and theranostics pipeline leveraging CRISPR/Cas. This figure summarizes the delivery of CRISPR components, such as exosomes/extracellular vesicles, nanoparticles, which occur *via* endocytic uptake, which can be receptor-mediated or receptor-independent, or *via* direct entry, such as electroporation or membrane diffusion. Upon entry, endocytic vesicles must avoid lysosomal degradation, in a process called lysosomal escape. Cytoplasmic as well as nucleus-localized CRISPR applications are highlighted, as well as optimization strategies benefiting from preclinical and clinical feedback, artificial intelligence/machine learning (AI/ML), and novel formulation for nanomaterials for delivery that improve bioavailability, efficacy, endosomal escape *etc.*

Theranostics, originally a term used in radiotherapy, combines both diagnosis and therapy for personalized treatment of diseases. CRISPR-based diagnostic tools have been generated to detect, for example, viral infections in patient samples with very high sensitivity. Meanwhile, CRISPR has revolutionized personalized gene therapy, targeting patient-specific genomic alterations to achieve successful disease treatment. This section discusses its contributions to disease diagnosis, CRISPR/Cas9-based Imaging, drug development, and the treatment of genetic diseases through genome editing, gene therapy, and epigenetic editing (Figure 3).

#### 3.3.1 Diagnosis

CRISPR/Cas has enabled scientists to develop more precise and efficient methods for detecting genetic mutations and diseases. Scientists have developed diagnostic tests that use CRISPR to detect genetic mutations in conditions such as cancer and HIV (Shademan et al., 2022; Uno et al., 2023). In these tests, the Cas9 protein is programmed to target altered DNA sequences associated with the disease. The presence of the mutation causes the sgRNA to direct the Cas9 enzyme to cleave the DNA, resulting in a detectable signal. For example, CRISPR has accelerated the detection of cancer biomarkers, such as shifts in specific microRNA expression profiles (miR-21 in breast cancer, miR-155, miR-17, miR-92a in lung cancer subtypes (Bagi et al., 2025), identify disease-specific point mutations [e.g., BRAF V600E (Etemadzadeh et al., 2024), EGFR T790M (He et al., 2022)], and identify viral DNA (e.g., human papillomavirus). By extension, this methodology has also enabled the tracking of mutated variants of viral DNA, such as spike protein mutations in SARS-CoV-2 (He et al., 2022). Recently, CRISPR-based biosensors have enabled tandem detection of multiple biomarkers (e.g., alpha-fetoprotein and miR-122 in hepatocellular carcinoma (Zhang X. et al., 2025), spike protein and nucleocapsid mRNA of SARS-CoV-2 (Zhang Y. et al., 2025). Dengue and Zika viruses have been detected by CRISPR-based reporter assays. Mechanistically, isothermal preamplification with Cas13 nuclease was used to identify viral origin single-stranded RNA (ssRNA) and introduce an array of enzymes and fluorescent reporter genes into cells. It has been shown that the matching Cas13 activates the cleavage enzyme and selectively cuts the associated fluorescent reporter gene when the CRISPR system detects the target gene, emitting a fluorescent signal (Gootenberg et al., 2018). Building upon this method, Ackerman *et al.* developed a platform named CARMEN in 2020 to identify pathogen infections, including SARS-CoV-2 (Ackerman et al., 2020). A fluorescently labelled reporter RNA, Cas13, and sgRNA are all included in the detection combination of this method. The reporter RNA attaches to the light-inactive fluorescent molecule, while the sgRNA recognizes a particular target nucleic acid (DNA or RNA) sequence. Similarly, a single-cell CRISPR strategy that utilized mCherry-tagged sgRNA insertion of known carcinoma genes (an HNSCC and SCC panel including genes such as *Notch1, Fat1,* and *Trp53*) was deployed in mouse epidermis to trace the proliferation of cells containing these tagged genes during tumour development. The strategy was to track the clonal expansion of these single sgRNA insertions using RNA-seq/sgRNA capture, and revealed that the tumour necrosis factor pathway as a whole is a major driver of tumorigenesis in skin carcinoma (Renz et al., 2024). Similar tools can be used in the future to study tumour heterogeneity and resistance mechanisms.

Another CRISPR-based platform, termed DNA endonuclease-targeted CRISPR trans reporter (DETECTR), used the CRISPR/Cas12a to identify HPV infections (Chen et al., 2018). The DETECTR system combined Cas12a ssDNase activation with isothermal amplification to detect nucleic acids with attomolar sensitivity. CRISPR/Cas12a (Cpf1) was utilized due to its capacity to produce targeted DSBs in the following system. The RNA-guided DNA binding triggers the selective ssDNA fragmentation activity of Cas12a, which ultimately leads to the destruction of both circular and linear ssDNA molecules. The reliable identification of a DNA target sequence that matches the 20-nucleotide guide RNA sequence with precision can separate closely identical DNA sequences, necessary for activating ssDNA cutting (Chen et al., 2018).

#### 3.3.2 CRISPR/Cas9-based imaging

Apart from diagnosis, CRISPR/Cas has found importance in generating imaging tools to visualize specific DNA sequences in living cells. This technology, also known as CRISPR imaging, uses a modified version of Cas9 fused to a fluorescent protein. The Cas9 protein is programmed to target specific DNA sequences, and the resulting fluorescence is visualized using microscopy (Van Tricht et al., 2022). In 2015, a genetic imaging technique that combines the CRISPR/Cas9 system was developed, named CASFISH, which stands for “CRISPR-associated split fluorescent protein *in situ* hybridization.” The following system uses a modified CRISPR/Cas9 system to target and bind to specific DNA sequences in a cell. When the Cas9 protein binds to its target DNA sequence, it triggers the recruitment of a fluorescent protein that can be visualized under a microscope. This technology can be used to identify the location of genes within specific cell types, investigate the regulation of gene expression, and study the spatial relationships between genes and other cellular structures (Deng et al., 2015; Ma et al., 2015). Another such technique is the incorporation of Pepper-stabilized self-degrading fluorogenic proteins, where Pepper-modified sgRNA prevents its degradation; therefore, Pepper-sgRNA represents sites with high-contrast subcellular imaging of genomic loci (Zhang Z. et al., 2024). Various other imaging techniques based on CRISPR/Cas9 have been developed, where modifications have been made to the sgRNA, the Cas9 protein, or both with a fluorophore. Additionally, multicolour imaging and a combination of PET imaging have been developed using the CRISPR/Cas9 system (Marciano et al., 2022). In summary, the advancement of CRISPR-based imaging platforms paves the way for real-time, high-resolution visualization of dynamic genetic processes across diverse biological contexts.

#### 3.3.3 Drug discovery

The use of CRISPR has been pivotal in two key steps of the drug development lifecycle: identifying druggable targets and identifying cellular drivers of drug resistance. Through the simultaneous screening of a multitude of genes, CRISPR screening has emerged as a key method for finding druggable targets (Miller et al., 2011; Makhov et al., 2020). The discovery of BRD4 as a potential target for cancer treatment is one instance of a successful therapeutic target discovery using CRISPR (Shi et al., 2015). BRD4 was eliminated from cancer cells using CRISPR, which slowed the development of tumours. BET inhibitors, a family of medications that target BRD4 and have shown promise in clinical studies, were developed following this discovery (Estoppey et al., 2021). Genome-wide CRISPR screens to identify regulators of drug resistance have gained momentum in recent years (Ipsen et al., 2022; Zhang B. et al., 2024). An innovative way to study drug resistance due to the function of a candidate gene is to systematically knock-in wild-type or mutant DNA sequences using HDR, and screen the cells with the drug itself. Such an approach has been termed DrugTargetSeqR (Kasap et al., 2014). A similar approach is a base editor screen, where a custom sgRNA library tiling the entire gene is paired with Cas9 base editors (C>T or A>G). Exposing cells to the candidate drug, which was transduced with a sgRNA library and a modified NG-Cas9, exposed its binding pocket in the gene, which, in this case, inhibits the inflammatory response of the cell towards bacterial lipopolysaccharides (Lampson et al., 2024). Such sgRNA-tiling screen paired with single-cell transcriptional profiling could uncover specific sub-domain regulatory elements in the candidate protein DOT1L that regulates resistance against anti-DOT1L therapy in a leukemia subtype (Yang et al., 2021).

#### 3.3.4 Treatment of genetic diseases

CRISPR/Cas9 technology has the potential to treat genetic diseases by correcting the underlying genetic mutations responsible for the condition. We present some techniques *via* which the CRISPR/Cas system has contributed to treating genetic disorders.

##### 3.3.4.1 Genome editing in mammalian cells

CRISPR/Cas9 has been used to directly edit the DNA sequence of the affected cells to correct the concerned genetic mutation. Leveraging CRISPR and HDR repair, ROS activity in phagocytic cells was restored by correcting a site-specific mutation in the *CYBB* gene, responsible for chronic granulomatous disease (Flynn et al., 2015). Base deletion has also been used to treat cataracts in mouse models (Wu et al., 2013). Restoration of the dystrophin protein in Duchenne muscular dystrophy patient-derived iPSCs (Li et al., 2015) and Atp1a3 in an alternating hemiplegia of childhood (AHC) mouse model (Sousa et al., 2025) are additional examples. CRISPR/Cas has also shown promise in managing HIV. A study reported that genome editing of CXCR4 and CCR5 using CRISPR-Cas9 prevented HIV-1 infection in CD4^+^ T cells by affecting the inhibitory receptors of T cells, like PD-1 and Lymphocyte Activation Gene-3 (LAG3) (Huang et al., 2017).

Chimeric antigen receptor T cell (CAR-T) cancer therapy involves engineering T cells to kill cancer cells by targeting tumour-specific antigens. During the production of CAR-T cells, the T cell receptor and MHC-I genes are knocked out by gene editing. To mitigate off-target effects of SpCas9, Madison et al. developed a dCas9-Clo051-based nuclease termed Cas-CLOVER, which utilizes both dimerization of the Clo051 nuclease domains and the utilization of double gRNA to drastically reduce off-target effects, thus yielding a high proportion of desirable T memory stem cells for effective CAR-T therapy (Madison et al., 2022). CRISPR/Cas9 technology has also been utilized to bolster the anti-tumour impact of CAR-T cells (Eyquem et al., 2017). A novel immunotherapeutic approach to treat cancer has also been developed using the CRISPR/Cas9 system by eliminating *SIRP-α* from macrophages, which improved their capacity to phagocytose cancer cells (Ray et al., 2018). Likewise, the CRISPR/Cas system has shed light on the discovery of treatment for various other potent genetic diseases like Hepatitis B virus infection (Wang D. et al., 2022), phenylketonuria (Villiger et al., 2018), transthyretin amyloidosis (Wen et al., 2022), and Alzheimer’s Disease (Bhardwaj et al., 2022).

As mentioned before, base editors are capable of making single-base replacements by utilizing Cas9 nickase activity and an enzyme such as cytidine deaminase, which enables C>T conversion in the nicked DNA. These have been especially useful in introducing non-synonymous gene mutations in specific sites. For example, CD45 is a ubiquitous pan-leukocyte marker that could potentially be an attractive target for CAR-T cell therapy, if not also targeting healthy hematopoietic cells. To circumvent this, a single substitution was made in the CD45 epitope that is recognized by CAR-T cells. This enabled their selective resistance, while successfully enabling anti-CD45 immunotherapy for acute myeloid leukemia, B-cell lymphoma, and acute T-cell leukemia (Wellhausen et al., 2023). Examples of different CRISPR classes with their applications in proteomics and theranostics is shown in Table 2.

**TABLE 2:** Different Cas systems and their example use in the fields of proteomics, diagnosis and, therapy.

Class	Type	Subtype	Effector	Target	Example of use in proteomics, diagnosis, and therapy
1 (multi-subunit Cas)	I	A, B, C, D, E, F, U	Cascade (CRISPR-associated complex for antiviral defense)	dsDNA	Rapid, instrument-free SARS-CoV-2 detection in clinical samples by Cas3 (Yoshimi et al., 2022) SARS-CoV-2 and influenza virus detection by Cas3 (Yoshimi et al., 2022) Cas3-engineered bacteriophage cocktail to reduce bacterial burden in chronic urinary tract infection
	III	A, B, C, D			SARS-CoV-2 detection from nasal (Kim et al., 2024b) swabs by Cas10 (Steens et al., 2021) SARS-CoV-2 detection by the type III-A complex TtCsm (Nemudraia et al., 2022) FIND-IT – a TtCsm-Cas13a tandem assay for one pot detection of SARS-CoV-2 (Liu et al., 2021)
	IV	A, B			
2 (single Cas)	II	A	SpCas9	dsDNAssRNA	Cas-CLOVER – dCas9-Clo051 to perform multiplexed gene editing in T cells (Madison et al., 2022) Del-2-Rec – a Cas9-based reactivation strategy of HbF in sickle cell disease (Felder et al., 2025) LNP-conjugated SpCas9 mRNA used for liver-guided delivery of transthyretin for in vivo editing (Gillmore et al., 2021) FLASH – detection of antimicrobial resistance genes in clinical samples by a Cas9-NGS method (Quan et al., 2019) GLoPro – a proximity labelling and quantitative proteomics pipeline for proteins associated with a specific genomic locus, targeted by dCas9 (Myers et al., 2018) CASFISH – multiplexed, *in situ* labelling of genomic loci (Deng et al., 2015)
			SpCas9 base editors (Cas9 nickase + deaminase)	dsDNA	VERVE-101 trial: a LNP-conjugated adenine base editor (ABE) deployed in vivo to inactivate PCSK9 in heterozygous familial hypercholesterolemia (Horie and Ono, 2024) BEAM-101 trial – ABE-based editing to initiate HbF expression in sickle cell disease (Base editing boosts hemoglobin in sickle cell disease, 2024) ABE employed to avoid off-tumour toxicity of CD45-containing CAR-T cells for pan-blood cancer immunotherapy (Wellhausen et al., 2023) CELLFIE – a CBE/ABE/dCas9-based platform to screen candidates that facilitate artificial CAR-T cell evolution to boost their efficacy in immunotherapy (Datlinger et al., 2025)
		B	FnCas9	dsDNA ssRNA	SARS-CoV-2 detection (Kumar et al., 2021)
		A	Cas12a (Cpf1)		DETECTR – a Cas12a-based nucleic acid detection platform (Chen et al., 2018) HOLMES – LbCas12a based fast detection of target DNA/RNA (Li et al., 2018)_ CASMART – a one-step diagnosis of cancer mutant alleles (Zhang et al., 2023)
		A	Cas13a (C2c2)		SHERLOCK – Cas13a-based nucleic acid detection (Gootenberg et al., 2017) CARMEN – multiplexed Cas13-based detection of 169 human-infecting viruses (Ackerman et al., 2020)
	VI	B	Cas13b (C2c4)	ssRNA	AuNP conjugated Cas13d reduced SARS-CoV-2 replication in vitro after a single administration (De Carli et al., 2025)
		C	Cas13c (C2c7)		
		D	Cas13d		

##### 3.3.4.2 Gene therapy

Building on the success of *in vitro* gene editing, CRISPR/Cas is showing strong potential in gene therapy treatments for various diseases. Gene therapy in animal models, in particular, has progressed rapidly along the translational pipeline. In one such example, *in vivo* editing of oxidation-sensitive residues in CaMKIIδ (Ca^2+^/calmodulin–dependent protein kinase IIδ) in the mouse heart offered cardio protection after ischemia-reperfusion injury, a major driver of cardiac disease (Lebek et al., 2023). Similar success has been seen for retinitis pigmentosa (Cui et al., 2024), humanized mouse model of prion disease (An et al., 2025), phenylketonuria (Rothgangl et al., 2025), and others. Recently, CRISPR cargo carrying systems have been developed to simultaneously monitor and administer gene therapy. Examples include treatment of an Alzheimer’s Disease mouse model by photoinducible system (Han et al., 2024), gold nanocluster (AuNC)-Cas9-gRNA conjugate for simultaneous monitoring and therapeutic editing (Tao et al., 2021), *etc.*

Several gene therapy approaches have seen clinical translation over the years. For example, sickle cell disease is a group of inherited disorders characterized by gene mutations that produce a sickling or crescent-like appearance of red blood cells, thus drastically reducing the ability of haemoglobin to carry oxygen. Scientists have used CRISPR to edit out a repressor, BCL11A, for foetal haemoglobin (HbF), the latter typically suppressed during development by the former. This is achieved by *ex vivo* editing of *BCL11A* in hematopoietic stem cells and bone marrow transplantation of the gene-edited cells to the patient. HbF re-expression restores the oxygen-carrying capacity of the sickle cell patient, and in a landmark decision, was the first CRISPR gene therapy approved by the US Food and Drug Administration in 2023 (Canver et al., 2015; Dever et al., 2016). A recent strategy to reactivate developmentally silenced embryonic and foetal globins uses CRISPR to delete the long intervening sequences that separate the strong β-globin enhancer from the distal HBE (embryonic) and HBG (foetal) promoters. Termed delete-to-recruit (Del2Rec), the strategy reduced sickling *in vitro* and could find future clinical applications (Felder et al., 2025).

Another milestone for CRISPR gene therapy is the recent successful personalized base editing treatment of a neonate with the rare genetic disorder carbamoyl-phosphate synthetase 1 deficiency (Musunuru et al., 2025). CRISPR has also been explored as a potential treatment for cystic fibrosis, a genetic disease affecting the lungs and other organs (Firth et al., 2015). Researchers have used CRISPR/Cas to target and silence the gene responsible for Huntington’s disease, a genetic disorder affecting the brain and nervous system (Monteys et al., 2017; Bjursell et al., 2018). By editing genes to restore vision, the CRISPR/Cas system has been explored as a potential treatment for retinal diseases, such as retinitis pigmentosa and age-related macular degeneration. (Du and Palczewski, 2023), hereditary angioedema (Longhurst et al., 2024), transthyretin amyloidosis (Gillmore et al., 2021), among others. While these examples illustrate the growing significance of CRISPR gene therapy approaches, it is not without potential adverse effects. For example, a Duchenne muscular dystrophy (DMD) patient treated with a high-dose AAV-packaged CRISPR transgene to restore dystrophin (the faulty DMD gene) expression led to an immune response and rapid death, possibly arising from a high level of accumulated vector genome (Lek et al., 2023). The outcome highlights the importance of careful scrutiny of CRISPR delivery vehicles in the context of human physiology.

##### 3.3.4.3 Epigenetic editing

Not only direct genetic manipulation, but it is becoming evident that inactive dCas9 may be employed as a DNA-binding platform to target fused epigenome-modifying enzymes, thereby altering the epigenetic state at a specific region (Nakamura et al., 2021). The catalytic core of human acetyltransferase p300 has been fused with dCas9, demonstrating that this system is sufficient for acetylating histone H3 lysine 27 at target locations and substantially activating target gene transcription (Hilton et al., 2015). In another such study, methyltransferases DNMT3L and 3A, together with the KRAB domain of ZNF10 (a transcriptional repressor), were fused to dCas9 to generate an epigenome-wide. Together with CRISPRon, a complementary dCas9-demethylase fusion protein, this approach enabled heritable epigenome-wide alterations without permanently altering the DNA (Nuñez et al., 2021). A similar strategy to reactivate foetal globin expression involved targeting methylation at the HBG promoter. In this study, the authors used CRISPR to disrupt UHRF1, a maintenance factor that regulates the methylation status of HBG by recruiting the methyltransferase DNMT1. In general, epigenome editing has emerged as a key strategy for beta-hemoglobinopathies (Bell et al., 2025). Taking a modular approach to epigenetic editing, researchers developed a chemically programmable CRISPR epigenome editor that fuses dCas9 with an FCPF motif, which recognizes and recruits small-molecule epigenetic editors to specific genomic loci. Using this system, JQ1, a chemical epigenetic silencer of *BRD4,* was successfully localized in the promoter region of *c-MYC*, an oncogene notoriously resistant to pharmacological targeting for silencing its expression (Altinbay et al., 2024). Overall, CRISPR/Cas9 has the potential to provide new treatments for a wide range of genetic diseases. However, much more research is needed to optimize the technology and ensure its safety and efficacy before it can be used widely in clinical practice.

## 4 Challenges and mitigation strategies

CRISPR/Cas holds promise for human therapeutics in treating genetic diseases, but there are also challenges that must be addressed. As the biotechnological and clinical implications of CRISPR/Cas become more apparent, so do the social and ethical concerns associated with its unpredictability. The use of CRISPR-based systems for altering the human genome presents significant challenges regarding delivery efficiency, off-target effects, and immunogenicity. To mitigate some of these challenges, short DNA/RNA sequences called aptamers are used, which can bind to specific intracellular DNA/protein targets. As such, aptamers can improve Cas9 specificity (Collantes et al., 2021), regulate Cas9 function (Kundert et al., 2019), subdue innate immune responses induced by Cas9/sgRNA delivery vehicles (Zhan et al., 2020), *etc.* Moreover, as stated before, the large size of Cas proteins presents a substantial obstacle to their packaging into viral vectors and *in vivo* delivery. Thus, there are ongoing attempts at Cas miniaturization with Cas12 and Cas13 proteins being at the forefront [Cas12f: (Ma et al., 2025), Cas12j2: (Pausch et al., 2020), Cas9d: (Yang et al., 2025), Cas13: (Zhao et al., 2023):]. In the context of gene therapy, there are ongoing attempts to develop advanced nanoplatforms, improving bioavailability, *in vivo* stability, targeted delivery, and endosomal escape, e.g., RNP-LNP (Hołubowicz et al., 2024; Chen et al., 2025), LNP-SNA (Han et al., 2025), lipid-coated inorganic NPs (LaBauve et al., 2023), photoinducible engineered exosomes (Han et al., 2024), biomimetic NPs (Wu et al., 2024) *etc.*

Another challenge is the occurrence of off-target effects when the sgRNA sequence matches a sequence other than the intended target sequence, which can potentially result in negative consequences. To mitigate this, researchers use Cas9 nickases and anti-CRISPR proteins. Very recently, scientists used large language models trained on a diverse set of proteins from the evolutionary tree and an engineered Cas9 with ∼400 mutations on wt spCas9 and demonstrated a stunning 95% reduction in off-target effects, termed OpenCRISPR-1 (Ruffolo et al., 2024). Artificial intelligence is also being used to increase on-target efficiency in online tools such as CRISPRon (Xiang et al., 2021), DeepCRISPR (Chuai et al., 2018), TIGER (Wessels et al., 2024), off-target prediction (Allen et al., 2019), prediction of outcome for prime (Mathis et al., 2025) and base editing (Kim N. et al., 2024), rational discovery of novel Cas subtypes (Feng et al., 2025), and enhancing the efficiency of existing editors by rational engineering (Park et al., 2025). With advances in natural language processing, it has now become possible to plan a fully customized end-to-end workflow for CRISPR experiments. Termed CRISPR-GPT, the large language model (LLM)-based system provides sgRNA design, selection of Cas and delivery methods, protocol, and troubleshooting (Qu et al., 2025). Given the unprecedented momentum of AI research, these tools will continue to evolve and have a greater real-world impact. Immunogenicity is also a concern, as Cas9 is recognized as a foreign antigen by the human immune system. Strategies to overcome this issue include using CRISPR/Cas in immune-compromised organs, administering it prior to birth, and engineering of immune-silenced Cas9 (Ferdosi et al., 2019), and usage of immune orthogonal orthologues of Cas9/AAV, which do not elicit an immune response in subsequent doses of gene therapy (Moreno et al., 2019). However, more research is necessary to establish CRISPR as a safe and effective therapeutic tool for human use.

Endogenous tagging to study protein-protein interaction and dynamics, as well as knock-in, are intrinsically tied to HDR efficiency. HDR can be biased by small molecule enhancers, such as RS-1 (Song et al., 2016), or the HDRobust pipeline, which inhibits DNA-PKc, NHEJ, and cell cycle restriction (Riesenberg et al., 2023).

Moving beyond technical challenges, ethical and regulatory hurdles remain, such as preclinical safety and clinical monitoring, concerns with human germline editing, informed consent for gene therapy, equitable access, etc (Guo et al., 2023; Wiley et al., 2025).

## 5 Conclusion and future perspectives

1. CRISPR has revolutionized the field of genetics and functional genomics by enabling precise genome editing and manipulation. However, the potential applications of the CRISPR/Cas system extend beyond genetics and into the realm of proteomics and theranostics. In the coming years, CRISPR is likely to have an even greater impact on the field of proteomics. One potential future perspective for CRISPR in proteomics is the development of CRISPR-based tools for targeted protein degradation. This approach could be used to selectively remove specific disease-causing proteins, leading to the development of new therapies for a wide range of diseases. CRISPR could further be used to modify proteins by increasing their stability or altering their enzymatic activity, which could pave the way for the development of protein-based treatments for a wide range of diseases, including genetic diseases, autoimmune diseases, and cancer.
2. Furthermore, the following technology can be employed to create novel diagnostic methods that identify protein-based biomarkers with high sensitivity and specificity, leading to the development of accurate diagnostic tests for various diseases. CRISPR is thought to be capable of being used in human therapeutics to treat genetic diseases by developing gene therapies to repair or replace faulty genes. This approach may lead to the development of novel treatments for genetic disorders.
3. The utilization of CRISPR-based systems can also facilitate the investigation of intricate interactions among proteins and other cellular constituents for a better understanding of protein functions and the identification of novel drug targets. In combination with other proteomic technologies, such as mass spectrometry and protein imaging, CRISPR could be utilized to better understand protein function and cellular processes. With new advancements in delivery vehicles, multiplexing strategies, epigenome editing, and AI-assisted engineering of Cas/gRNA design, CRISPR has moved beyond a geneticist’s toolbox and started to show real-world impact, as evidenced by recent approval of CRISPR-based gene therapies. Going forward, we may see many such successful clinical translations.

In summary, future advancements in these domains are expected to be shaped by:

CRISPR-integrated proteomics: emerging CRISPR modalities, including proximity labelling and targeted protein perturbation are anticipated to enable high-resolution mapping of protein networks and post-translational modifications, facilitating functional proteogenomics.

Theranostic applications approaching translation: CRISPR-based nanoplatforms that co-deliver gene editors with imaging probes or therapeutic payloads are progressing toward clinical evaluation, enabling real-time monitoring and personalized treatment.

Convergence with AI, nanotechnology and single-cell omics: AI–assisted Cas/gRNA design, nanocarrier delivery and single-cell multi-omic profiling will only accelerate precision engineering of CRISPR systems and their application in patient-specific diagnostics and therapy.
